# “Believe me, only I know how I feel.” An autoethnographic account of experiences of epistemic injustice in mental health care

**DOI:** 10.3389/fpsyt.2023.1058422

**Published:** 2023-02-23

**Authors:** Lill Hultman, Maya Hultman

**Affiliations:** ^1^Department of Social Sciences, Marie Cederschiöld University, Stockholm, Sweden; ^2^Independent Researcher, Stockholm, Sweden

**Keywords:** epistemic (in)justice, in-patient mental health care, disability and mental health, personal assistance, critical personal narratives, Sweden

## Abstract

In Sweden, support and service for people with disabilities is provided under the Swedish disability legislation, which has a clear focus on the individual’s right to a life like that of any other citizen and on promoting equality and participation in society. Nevertheless, having a physical impairment makes it clear that equal mental health care is not provided in practice. This becomes particularly salient when there is a need for mental health in-patient care. In this article, the aim is to explore our own experiences of epistemic injustice in relation to mental health care provision in a situation where one of us has a mobility impairment that require the presence of personal assistants in everyday life. Critical personal narrative is applied to highlight the different, but intertwined experiences of a young female mental health user with a physical disability and her mother. Diary entrances, shared discussions and extracts from health care records are used to illustrate how epistemic injustice may occur in health care practices. In the analysis, we use Fricker’s concepts that relate to different aspects of epistemic injustice, to show how power is exerted. Healthcare professionals’ inability to value and integrate patients experience-based knowledge into practice where the lack of a holistic perspective visualizes what happens when people do not fit into predefined categories. Instead of strengthening patients’ rights, health care professionals discredit patients’ and family members knowledge, and thereby giving themselves epistemic privilege. People with the combined experience of both disabilities and mental health issues are vulnerable to epistemic injustice and epistemic harm since they are commonly denied both epistemic credibility and authority. Our results highlight the importance of counteracting resilient structures of social privilege and power and identifying and, in as far as possible, removing the mechanisms that exclude the epistemic resources of people with disabilities and their family members from being part of shared epistemic resources.

## Introduction

In Sweden, support and service for people with disabilities is provided under the Swedish disability act, Act concerning Support and Service for Persons with Certain Functional Impairments, LSS. Swedish disability legislation has a clear focus on the individual’s right to live a life like that on any other citizen, with an emphasis on promoting equality and participation in society. If people due to disabilities need support with care needs such as “… personal hygiene, meals, dressing and undressing, communication with others or other help that requires extensive knowledge about the person with a functional impairment” (1), they are given access to some degree of personal assistance. Personal assistance is an individualized support that entails user control (2) and is to be provided by a limited number of people (personal assistants).

In Sweden personal assistance is regulated by two different pieces of legislation- the LSS Act- and the code of statues. To obtain personal assistance, the person with disability must identify and describe the need for support and make an application. Then either an LSS-officers at the municipal level or officials at the Social Insurance agency at the state level, are responsible for conducting the social investigation that decide if a person can gain access to personal assistance. Psychiatric services are provided by the regional health care system, and include inpatient treatment, medication and outpatient care. Because of this division of responsibility between disability support and psychiatric support, there is a continuing challenge to (3) coordinate interventions from different service providers (4).

The consideration of basic rights is important from both a perspective of non-discrimination and for supporting full participation in society (5). The UN Convention on the Rights of Persons with Disabilities (UNCRPD) (6), which came into effect in 2008, is considered one of the main reasons for a shift in thinking about disability from a social welfare concern to considering a human rights issue (7). Social justice is central to the concept of equality. Viewed from a disability perspective, a prerequisite for social justice is equal participation in society. However, people with disabilities suffer both socioeconomic injustices, such as deprivation, and cultural injustices, such as non-recognition and disrespect (8). According to crip theory (9) a person’s ability is fluid. Nevertheless, our ability is understood as normal or deviant in relation to how well it follows notions of compulsory able- bodiedness. The concept of able-bodiedness is a culturally compelling expectation, which implies that it is both taken for granted and considered as aspirational (9). Ableism affects people with disabilities opportunities to participate as well as identity formation, self-understanding, and self-worth. It affects both societal design and cultural beliefs.

In culture and the media people with disabilities are often stereotyped, and cast either as victims and objects of pity, or heroes and inspirational role models for overcoming their impairment (10). Disability discrimination is evident in the culture, both in terms of either lack of representation or undifferentiated representation in media, where people with disabilities are subjected to stereotyping (11). When societal or institutional patterns of cultural and symbolic value construct people as inferior, or just invisible, there is a lack in full partnership in social interaction and hence a state of misrecognition (8). In terms of identity, recognition and redistribution might be constructed as mutually exclusive, but from a status point of view they become integrated (8).

For many people with disabilities, their mental or physical issues play an important part in their sense of self. Thus, it becomes important to have a holistic approach to disability and understand disability as a dynamic interrelationship between an individual with a health condition and the environment in which they find themself (12). Already in 1977, the World Health Organization advocated for patients to participate in their healthcare (13). A patient-centered perspective requires that health care professionals holistically consider what is known about a patient and understand the patient as a unique human being before determining a diagnosis (14). The purpose of the UNCRPD is to promote, protect, and ensure the full and equal enjoyment of human rights and fundamental freedoms by all persons with disabilities and to promote and respect their inherent dignity. Article 4 in the UNCRPD clarifies that state parties should take all appropriate measures to eliminate discrimination based on disability. Nevertheless, mental health is unequally distributed depending on a range of discrimination grounds: gender, country of birth, disability, and sexual orientation (15). Persons with disabilities consistently display a higher prevalence of mental ill health (15, 16). A deterioration in mental health appears to have occurred over time among young people with disabilities (16). Between the years 2017 and 2020, the number of patients in Swedish child and youth mental health care increased by 13 percent (+15,800 patients) and the number of visits to psychiatry units increased by 11 percent (+110,000 visits) (17).

In this article, the aim was to explore our own experiences of epistemic injustice in relation to mental health care provision in a situation where one of us has a mobility impairment that require the presence of personal assistants in our everyday life.

## Epistemic injustice

Epistemic injustice describes a situation where certain types of knowledge are not taken seriously for understanding, interpreting, or defining a situation. Epistemic injustice involves certain people being subjected to knowledge-based discrimination based on an attributed deficit of credibility in relation to possessing knowledge. Being subject to epistemic injustice makes it difficult for situated knowers to make sense of their own experience or understand what is in their best interest to know (18).

Fricker (19) distinguishes between two types of epistemic injustice: testimonial injustice and hermeneutical justice. Testimonial injustice occurs when hearers due to prejudice undermine, exclude or dismiss persons in their capacity as potential knowers. It also impedes speakers from expressing critical thoughts and ask critical questions. In addition, repeated transgressions can result in speakers staying quiet in situations where they should not, for fear of further marginalization. Hermeneutical injustice occurs when one-part lacks words or expressions to make themselves understood in a specific context or situation, and in which their own or others interpretative resources puts them at a disadvantage when trying to make sense of their experiences (18).

Even though epistemic injustice is enacted in micro-meetings these harmful actions often derive from epistemic practices which can be found on a structural level (18). Hermeneutical injustice is not committed by a single entity, “but is caused by a particular aspect of our collective hermeneutical resources: either an individual gap (in the temporary case) or a more extensive deficiency caused by structural identity biases (in the systemic case)” [(19), p. 231].

In the “credibility economy,” the resources of credibility – knowledge and access to different concepts – are unevenly distributed between different individuals and social groups. This uneven distribution of resources can lead to hermeneutical marginalization, which implies that a socially disadvantaged group (19), such as people with disabilities, is blocked from gaining access to knowledge or communicating messages to more socially privileged groups (19). In epistemic exclusion, certain kinds of knowledge are not included in the shared knowledge bank (20). People are epistemically excluded when they are unable to access epistemic resources and/or contribute to the generation thereof. Epistemic privilege is enjoyed by dominant groups in society, since their forms of knowledge are preferentially absorbed into the epistemic resources that make up the background knowledge of a given community (21). Injustice arises because it systematically advantages certain parties, such as the health sector, at the expense of others such as marginalized groups and communities (22).

## Materials and methods

Critical Personal Narratives (CPN) is used as the research methodology (23) to highlight the different, and sometimes intertwined narratives of a young, disabled, female mental health patient and her mother. In, CPN, also known as critical autoethnography, personal experience is used to criticize, analyze, unsettle and defamiliarize what is often passed off as the ordinary, or the routine.

Autoethnography is used as a tool to describe and deconstruct power relations and marginalization (24). Mutual experiences of living a life where political decisions, debates in the media, and bureaucratic decisions constitute a potential threat to one’s way of life are an ongoing trauma, as described by Ryan (25), which is a reality that the two authors share and are forced to deal with.

### We, as mother and daughter

In this study, our dual roles of the researchers and the participants of the study at the same time feature intensively and throw an issue of reflexivity of qualitative research. We both have similar and very different experiences of encounters with health care staff involved in provision of care. We are bounded by our mutual experiences of numerous encounters with a variety of health care professionals such as: psychiatrics, physicians, nurses, mental health nurses, and assistant nurses. We also have a mutual engagement in a local disability organization for families and children with mobility impairments. Nevertheless, our experiences differ in significant ways, both in relation to our respective roles (mother and daughter/recipient of personal assistance and psychiatric care) and in relation to our embodied experience (disabled/non-disabled person). One of us- the daughter is a young woman with a mobility impairment, in the beginning of university studies, transitioning from child and youth care to adult care. The other one- the mother is a middle-aged, non-disabled woman, with a background as a social worker and disability researcher. Sharing household, our lives are interconnected by mutual experiences of being in a vulnerable life situation, where access to personal assistants set the boundaries for our participation in society.

Living together creates opportunities for in-depth discussions about sensitive topics, knowing each other well makes it easier to be candid and being familiar with the situations referred to on a more detailed level give us a unique opportunity to provide two different perspectives on the same situation. In addition, being family members could make us influence each other’s narratives. Being mother and daughter can undeliberate make us assume that we know each other’s perspectives which could lead to not asking clarifying questions.

One of the biggest challenges of using CPN in studying disability and society is that we used our subjective experiences and feelings in the research. We may have some biases or personal experiences that are different from the experience of others. We treat this subjectivity as an approach to understanding our ways of knowing while exploring the issues of psychiatric health services.

Due to the emotional content of the text, it has been necessary for us to rest from the text for periods of time. Events narrated have been selected to illustrate critical incidents involving different actors in psychiatric care, at in-patient care units and out-patient care facilities. When care units are referred to in this text pseudonyms are utilized.

A third person perspective is utilized to critically analyze and reflect on our narratives. This gave us an opportunity to understand our narrative data and rethink the issues with a more objective point of view. Therefore, the daughter is called Amanda and the mother is called Anna.

We did not go through the application for ethical review because we did a textual-based analysis through our personal narratives. As authors and participants, both of us agreed to share our personal reflections and thoughts in this research.

The analysis began with the second author identifying critical incidents. Based on these incidents we discussed our experiences and the meaning and relevance in relation to access to equal care for people with disabilities. The second step was to complement our own narratives with notes from anonymized hospital records made by psychiatrists in charge of Amandas care. The third step was to create themes based on the chosen incidents and analyze them deductively by utilizing some of the core concepts that unpack the mechanisms of epistemic injustice, such as Fricker’s (18, 19) concepts, testimonial injustice and hermeneutical injustice, which underline how power is exerted in a mental health care context by different care providers. In addition, to further clarify the relationship between disability, mental health, and epistemic injustice, concepts developed by the critical disability scholar Garland Thomson concerning misfitting were used (26, 27).

## Results

The results are based on the themes discovered: “Believe me, only I know how I feel,” Health care staff’s reluctance to provide for basic care needs, and health care professionals perpetuating their epistemic privilege.

### “Believe me, only I know how I feel”

Within adult psychiatry, many of the out-patient psychiatrists and unit managers for in-patient care held beliefs that in-patient care is not the right place for Amanda, even though she finds it necessary to be admitted to an in-patient care unit. The chief psychiatrist tried to persuade her that it was better to stay at home and cope with her ordinary out-patient care interventions. Although the chief psychiatrist responsible for Amandas care knew that the treatment was not effective, she was not willing to adjust the treatment plan. Due to experiences of severe anxiety, suicidal thoughts and impulses to self-harm, out-patient psychiatrists often agree to admit Amanda to an in-patient care unit. Nevertheless, those decisions are often questioned by chief psychiatrists at different in-patient care units that believe that in-patient care is not the right place for her, which result in Amanda being discharged although she has told them that she still has thoughts about self-harm. Particularly one of the chief psychiatrists at one of the in-patient care units routinely dismissed Amanda’s testimony of suicidal thoughts and self-harm. Amanda consequently suffers testimonial injustice. Since both psychiatrists in outpatient and in-patient care foremost categorized Amanda as a disabled person, prejudicial stereotypes of disability and disabled people hindered them from listening to Amanda and taking her seriously.

A common denominator for the psychiatrists was their willingness to tell Amanda what they thought her mental health issues derived from. They said that her mental health issues were due to communicative difficulties within her family and lack of access to enough personal assistents. One of the psychiatrist responsible for inpatient care at one of the care units made the following assessment in the medical records:

The patient appears calm and adequate in the ward, does not suffer from a serious mental disorder, is not depressed, not psychotic. A difficult home situation increases the patient’s instability and negative thoughts. (Medical record entry from a psychiatrist, at an inpatient care unit, December, 2021)

In another entry in the medical record, from the same care occasion, the psychiatrist writes:

18-year-old woman discharged to her home and habitual state. Came in due to a burdensome social situation. (Medical record entry from a psychiatrist at an inpatient care unit, December, 2021)

In medical record entries made in connection with discharge from psychiatric inpatient care and follow-up in outpatient care, the psychiatrist in charge writes:

In summary: young woman with cerebral palsy as main diagnosis. Anxiety and destructive behavior in connection with stress. Personal assistant during waking hours. (Medical record entry from psychiatrist at an inpatient care unit, December, 2021)

Eighteen-year-old female with psychiatric diagnosis but mainly cerebral palsy who has come in for a check-up after discharge from *Gullvivan* [name of inpatient ward]. Received good care there. (Medical record entry from psychiatrist, outpatient care, January, 2022)

Instead of validating her experience of poor mental health, irrespective of origin, the psychiatrists chose to focus on her disability and the lack of adequate social support interventions, which they considered to be the main cause of her suffering.

It is strange that health care professionals give themselves the right to define what the main diagnosis is and that representatives of psychiatric care focus on writing that the main diagnosis is cerebral palsy (the functional impairment), regarding which they have neither knowledge nor treatment responsibility.

I am a whole person, neither just a body nor just a mind – I am so much more. There’s nothing wrong with me, I’m not a defective person, although health care usually describes me as sick or broken. A neurologist at the children’s and youth clinic used the word “defect” to describe how much mobility I have in my elbow joint; she wrote that I have an “extension defect in the elbow joint up to 20–30 degrees” (Amanda).

When Amanda attempted to describe her everyday life situation from her perspective, psychiatrists attributed her a credibility deficit based on her descriptions of her overall life situation. Instead of trying to understand the complexity of her everyday life situation, the responsible psychiatrists seemed to pay attention to those narratives that resonated with their preconceived perceptions about who belong in an in-patient care unit and benefit from psychiatric care. Accordingly, they communicated that Amanda’s emotional distress would be manageable if she had access to either independent living with personal assistants or were placed in a service home with round the clock staff. By recasting and reducing Amanda’s mental suffering to consequences of her disability, psychiatrists not only gave themselves epistemic privilege, they also caused epistemic harm by silencing Amanda and stripping her of agency (18).

In contact with mental health care, both Amanda and her mother, Anna, has learned that it is not enough for Amanda to say that she has suicidal thoughts. There must be visible, objective evidence of self-harm. The absence of visible injuries is considered “proof” that Amanda can deal with her mental health condition and is used to discredit the patient’s verbal account of mental suffering and classify it as manageable. The outcome of Amanda’s clinical assessment also depended on which psychiatrist was on duty at the time and day in question, which assessment unit that psychiatrist belonged to, and the availability of inpatient care. Amanda recalled a conversation at the in-patient care unit when she still belonged to child and youth mental health care,

At the BUP (child and youth mental health care) emergency unit, the psychiatrist asks me to describe how I feel, and I begin. She listens and takes notes. She then explains that there are no openings that evening, but that my condition is serious. I keep saying that I feel very bad, and I state this repeatedly. Then she asks to see my arm, asks me to roll up my sleeve. She asks if I can do it myself. I declare that I cannot [roll up my sleeve] and cannot harm myself so that it shows. I can’t seriously injury myself physically, but in these moments, that’s all I want. My body does not obey, and therefore I can only injure myself superficially. My thoughts are just as destructive as those of a self-harmer.

When Amanda was admitted as a patient to an adult in-patient care unit, she had learned that it was important for her “to prove” that she was ill enough – otherwise there was a risk of her being discharged while thoughts of self-harm and suicide still remained. Amanda reflected:

I’m sitting here with a lot of anxiety and have been thinking about hurting myself for 20 minutes. In the end, I do it mostly because I’m sad and lonely. I ring the red bell and, strangely, I expect it to be like in a casino – that something funny happens when you pull the red lever, but all that happens is that a bored assistant nurse comes in.

The assistant nurse asks what I want in a hostile manner. I reply that I have harmed myself and that they should know about it. He says in a disinterested voice: “Show me.” I show the small wound that I have scratched on the back of my hand, over the scars that reveal all the times I have scratched myself before. The assistant nurse says “Stop it, don’t do that” and leaves the room. I know it’s just that the wound is small and looks insignificant, really. But half to annoy him and half to get help, I ring the alarm again and hope not to meet the same assistant nurse again. Of course, the same tired face comes back through the door. He says: “What now?” “Yes, but the wound, aren’t you going to do something about it?” “What?” “Yes, but I’ve harmed myself, aren’t you going to clean it?” He cleans the wound, irritated – half because I want him to and half so that I won’t call him again. “Don’t do that again,” he says, and it just feels like the same scene is playing out over and over.

### Health care staff’s reluctance to provide basic care needs

When Amanda expressed that she needed to utilize the toilet or eat breakfast, she knew that it would probably make the staff feel stressed and uncomfortable, which created a strained relationship that might affect her treatment. Although, assistant nurses and mental health nurses were less likely to ask questions, some of them showed their discontent and disbelief while helping Amanda. Not wanting to be perceived as a nuisance, Amanda found it difficult to ask for help:

They will say something like “You’ll have to wait, we are only a few assistant nurses in the care unit now. We’ll come by as soon as possible.” Then it takes anything between 15 and 30 minutes before someone comes. Sometimes they forget that they can’t assist me on their own and then it takes another 15 or 30 minutes. It becomes even more difficult to decline help from male staff when I have been told that there is no other solution. Being upset when declining help can cause you to be perceived as a troublemaker, which can justify staff using forced medication in the form of sedatives. You can be labelled as “difficult,” “uncooperative”, or “unruly.”

Psychiatric staff members were not used to provide physical care and some of them lacked a formal assistant nurse education. Both assistant nurses, mental health staff, and nurses lacked knowledge about mobility aids and expressed feelings of uncertainty when utilizing them. Thus, some of the assistant nurses and other mental health staff were reluctant to provide care and handle assistive devices. In addition, this was not among their regular duties, and when there was shortage of staff, it became hard for them to both perform their regular duties and function as personal assistants. Amanda perceived that interactions between her and some of the assistant nurses became tense since they displayed fear and pity towards her. Medical records also confirmed that having to perform tasks that was not considered as part of their ordinary duties created dissatisfaction among staff, which was expressed in the following journal entry:

The undersigned [psychiatrist at outpatient care unit] has been in contact with the chief psychiatrist at *Gullvivan* [name of the inpatient care unit] and it appears that the patient has no assistance at the ward there and that the ward staff are not trained to be personal assistants to the patient. This has created negative sentiment among the staff against the patient. (Medical record entry from psychiatrist in outpatient care, December, 2021)

Amanda’s primary reflections when she read the record entries were that they confirmed what she felt during her stay at that unit, where she experienced the interaction with mental health care staff as being tainted by her visible disability. Reading also made Amanda sad and distressed regarding future needs for inpatient care, since mental health care staff and managers at *Gullvivan* showed no ambition to make their care facilities more accessible neither in relation to psychosocial treatment nor as regards the physical environment. Amanda reflected:

It feels difficult, reading that health care professionals find it problematic to help me. It makes me feel singled out and responsible for solving their problems. They often complain in front of me, which makes me agree to solutions that don’t feel good for me. The environment also contributes to me feeling like a problem. The premises are not adapted for people with physical disabilities: the rooms are small and there is no space for my aids. None of the patient rooms are adapted for wheelchair users. Sometimes, this means four people will try to do a joint lift, where I am moved from my wheelchair to the toilet. If these four people do not communicate clearly with each other, the lift becomes risky, it feels uncomfortable, and I end up sitting crookedly on the toilet. The communication between me and the staff reduces me to a body or an object, to be moved from one place to another. No one thinks to ask me how it feels or what would work. If I get a question, it’s if I’m okay with male staff helping with the lifting. If I say no, then there will be four women lifting my body, instead of a “strong man.” It is difficult not to feel like a problem, which makes it even more difficult to say no to the help offered, in a situation that does not have an obvious solution and where both staff and patient end up in a deadlock.

As Amandas mother, Anna found it difficult to stay balanced, and not become too upset when she realized that Amanda did not receive proper care at in-patient care units. Sometimes Anna thought that her background as a disability researcher enabled her to stay calm and analyze her and Amandas encounters with health care staff as well as the health care system. At other times she felt that having knowledge about disability legislation and recognizing the discrepancy between law, policy documents and practice made her feel even more frustrated. Anna reflected:

I easily, become frustrated with the Swedish health care system that cannot help Amanda who have complex care needs. It is obvious that patient-centered is one of those magic concepts and that equal care does not apply to her. When I went to visit her at the in-patient care unit yesterday, I instructed two assistant nurses in how to use Amandas assistive devices, so they could show their colleagues. The next day, Amanda tells me that it did not make any difference, and the routine is back to getting help from four female staff members or the care unit’s “strong man.”

### Health care professionals perpetuating their epistemic privilege

When Anna or Amanda told health care staff that decisions made by officials at the Social Insurance Agency do not allow personal assistants to work when Amanda is admitted to in-patient care, they were met with disbelief. Although chief psychiatrists and unit managers did not tell them that they were wrong, they conveyed their disbelief through questions and advice, such as: Why do not you apply for assistance during hospital stay? or have you had any contact with the municipality? They can provide disability support.

A journal entry made by a one of Amanda’s psychiatrists exemplify health care professionals lack of knowledge about other authorities and care providers responsibilities:

The mother informs us that the Social Insurance Agency has decided that the patient does not have the right to assistance when she is admitted to health care facilities. The undersigned [the chief physician] is a little surprised and explains that staff in the psychiatric department are not used to providing physical assistance and that the company providing the assistance should have an agreement to do so even when the patient is admitted. (Medical record entry from psychiatrist, outpatient care, December, 2021)

In this situation, the chief psychiatrist lacked knowledge about current implementation of Swedish disability legislation and the process of gaining access to personal assistance. She stated that we must demand that our assistance company provided access to personal assistance during inpatient care. Despite explanations on our part, psychiatrists’, and other health care professionals at different both in-patient and out-patient care units insisted that we must understand that care staff were not able to replace personal assistants. Some of them were under the impression that we did not understand the working conditions of care staff and thus informed us of a situation that although it was well-known to us, we did not find acceptable.

The negative consequences of needing support from both health care and social services becomes particularly salient when the provision of support was affected by decisions and guidelines from different authorities and care providers that were unaware of each other’s competence and responsibility (4). Although this situation was familiar to Anna, she became both angry and frustrated since there was no single person from who to demand responsibility:

The experience that stays with me is that the existence of a complex life situation is used as an excuse for health care providers to try to limit their responsibility and transfer it to the municipality – which is supposed to solve the situation, because there is no “mental illness.” Everything takes time, time that we don’t have. What happens when we can’t take it anymore?

Over the years, Amanda and Anna have had numerous meetings with different welfare actors, where both Amanda and health care staff has given Anna the main responsibility for coordinating Amanda’s care interventions. Anna has had this role since Amanda was granted personal assistance for the first time. Amanda was 6 years then and is 19 years old now. Anna often reflect upon the difference between making a phone call as a professional health social worker or researcher, versus making it as a private person. She possesses the same knowledge, but her input has been given different value when she is cast in the role as a professional. Even though she has had access to epistemic resources such as hermeneutical tools (medical discourse, familiarity with hospital work, LSS-legislation, social work), this was not sufficient to equalize existing power structures when she is viewed “only a parent,” which downplayed the relevance of her combined experience-based and professional knowledge. Anna felt taken advantage of by health care staff as she was always expected to show up and be available when the health care staff thought she should participate in care planning and other care visits. Even though health care professionals considered Amanda’s mental health issues was due to communication problems with her mother, they expected Anna to take responsibility for coordinating Amanda’s various care efforts:

As a parent, it feels like a moral obligation and an expectation from health care staff that I should always be available. When health care professionals feel reluctant to shoulder responsibility, representation is not questioned – then I am expected to act as an interpreter, mouthpiece, and representative. In other circumstances, I can be perceived as a potential threat, either depriving my daughter of her voice or speaking in my own interest. As a mother, I am expected to be there when it is convenient for health care providers.

Instead of seeking collaboration and shared responsibility with other care providers, health care professionals expected patients or parents to coordinate support and care interventions. Lack of knowledge about other authorities’ or caregivers’ areas of responsibility, combined with insufficient knowledge about the individual patient, made coherent care planning difficult and created a situation where the responsibility for coordination of support was placed on family members and the person in need of support. Thinking about her lifelong needs of care, increased Amanda’s anxiety and she often got stuck with negative thoughts about an imagined future where she has to stand up for herself without the support from her mother:

What happens when mum no longer is there to pick up the pieces of the broken healthcare system? A system that tells me that I don’t belong there. That I would be better of living in an assisted living facility. Somewhere else where I no longer have to worry about being without help. Where they are better equipped to look after someone like me, they say. How can they even say that, when they don’t know the first thing about me or even people like me cause I’m the first one they have ever met with a physical disability. Who will make sure my rights are still intact, and that I get a say in what I need from health care staff?

By assuming that Amanda and Anna were ignorant of the roles and duties of health care staff at inpatient care units, health care professionals simultaneously discredited their capacity as knowers while expecting Anna to take the main responsibility for coordinating social support with different health care providers. This line of reasoning places both the blame and the responsibility for the problem on patient and relatives. In addition, the psychiatrists did not question their own expertise. Even in situations when it was obvious that psychiatrists were misinformed, they did not take the chance to learn something new, instead they chose to retain their epistemic privilege.

## Discussion

In this article, the aim was to explore our own experiences of epistemic injustice in relation to mental health care provision in a situation where one of us has a mobility impairment that require the presence of personal assistants in our everyday life.

In the backdrop of austerity politics (28), having a physical disability that requires technical aids as well as personal assistance reveals that equal health care does not, in practice, extend to people with severe disabilities that require both somatic and mental health care interventions. Although it is the duty of the psychiatry unit to provide equal and patient-centered care, the encounter indicates epistemic injustice that according to Dotson (29) derives from epistemic systems, from which individuals may be excluded to greater or lesser extent. As a stark contrast to having an intersectional approach, mental health care professionals tend to focus at one intersection at the time, and almost exclusively on Amandas disability.

This becomes particularly salient when there is need for mental health inpatient care. In mental health care facilities, rooms are usually inaccessible for people with physical disabilities. When Amanda needs space to move around with her wheelchair or utilize the bathroom, the environment needs to be adapted, e.g., furniture must be moved, which both staff and managers find difficult to do. This recurring task frames disability and the disabled person as problems that need to be fixed (30). Garland Thomson (26) has conceptualized this as the concept of a misfit or a situation of misfitting; “People with disabilities become misfits not just in terms of social attitudes—as in unfit for service or parenthood—but also in material ways. The disadvantage of disability comes partly from social oppression encoded in attitudes and practices, but it also comes from the built and arranged environment.” [(26), p. 594]. It underlines that “the discrepancy between body and world, between that which is expected and that which is, produces fits and misfits” [(26), p. 593]. In line with the reasoning of Garland Thomson (27), the disability dominates and skews the perceptions of non-disabled people, meaning that they tend to reduce a disabled person’s complex personality to a single attribute, i.e., disability.

To misfit in the public sphere is to be denied full citizenship (26), since equal access to the public sphere – which include institutions such as health care facilities – is denied. When personal assistance is not granted for inpatient care, the lack of an accessible toilet (*misfitting*) is transformed into an individual problem, where the situation defines the person – who thus becomes a problem (*misfit*) that health care workers are expected to solve. Without the support of managers, nurses and assistant nurses are forced to find a quick solution to a structural problem. When this happens, there is a great risk that health professionals’ frustration is transferred to the patient, who becomes the scapegoat. Foucault (31) describes self-discipline as something that occurs everywhere in society where power is exercised – whether expressly stated and implied. At first glance, self-discipline can be perceived as the individual wanting to subordinate themself. When individuals discipline themself, they adapt to what the environment wants without any external pressure being needed, which means that the demands of the external power move into us and we are disciplined into subordination to get what we need (32).

Health care professionals’ inclination to mistrust and devalue experience-based knowledge provided by people with experience of disability and mental illness or their family members contributes to testimonial injustice, which sustains the epistemic injustice whereby a significant area of knowledge is obscured from the collective understanding [(19), p. 154–155]. Thus, epistemic harm occurs both in relation to lack of adequate support with basic care needs in inpatient care and more indirectly by silencing the experience-based knowledge and devaluing its worth.

Instead of strengthening patients’ rights, which could be reinforced and further developed by utilizing the experience-based knowledge that resides in people who have lived experiences, health care professionals dismiss their knowledge, thus giving themselves or other type of knowledge sources an *epistemic privilege*. As pointed out by Fricker (19), testimonial injustice can operate through individual actions and responses. Although we have both communicated – to several people belonging to different mental health care units – that the authorities’ current decision on assistance for Amanda does not allow personal assistants to work when she is admitted to inpatient care, this is interpreted as our having misunderstood our rights to get support or having failed to seek adequate support from the authority in charge. This is a typical example of testimonial injustice, in which our experience-based knowledge is dismissed, perhaps because it is assumed that the information we provide (as a disabled person and an ally) is inherently untrustworthy in a way that information provided by health care staff is not. As a disabled person, Amanda is vulnerable to epistemic injustice. People with disabilities are commonly denied both epistemic credibility and authority (33).

When Amanda communicates her needs of assistance with basic care to staff at inpatient care units, they do not give her time to explain how she wants to be supported – instead they make decisions regarding how and when support will be provided. In those situations, health care staff – in addition to treating her as a problem (*misfit*) – give her an undifferentiated response: she is not seen as an individual with a physical disability, but as a member of the group “disabled people,” irrespective of her individual circumstances. Since this type of testimonial injustice is inflicted systemically, it is even more damaging and insidious than if it were experienced at the hands of an individual (18, 19, 33, 34). Having claims and accounts epistemically downgraded unsettles a person’s trust in the epistemic value of their own narratives and judgments and can, if internalized, impair their confidence in their overall agential capacity. It can also create a feeling of hopelessness and despair. Scully (33) argues that this becomes particularly harmful to disabled persons, who often have their status as people with the same moral value as others called into question.

Even in situations when health care professionals or civil servants agree with patients and family members, there is rarely any difference in practice, since the problem is recognized and described as a systematic error at a structural level, for which individual practitioners, civil servants, and organizations cannot take responsibility. Professionals’ inability to value and integrate patients’ experience-based knowledge into practice (35) becomes particularly salient in the transition phase from child to adult care, where the lack of a holistic perspective reveals what happens when people do not fit into the predefined categories of either welfare recipients or health care users. A complicating factor is that care is administered and shared between different authorities and organizations, with decisions made and enforced through different legislative acts and at different levels of governance (state, regional, and community level).

One of us is holding a faculty position as a disability researcher which has enabled us to have the opportunity to publish our article in an academic journal. We acknowledge this fact, as an epistemic privilege compared to other people whose lived experience remains untold due to lack of both financial and hermeneutical resources such as funding and knowledge about academic language and writing processes. Nevertheless, we believe that utilizing our epistemic privilege is justified in the sense that it enables us to provide an inside perspective on issues of epistemic injustice that need to be addressed. In this text health care professionals are not portrayed in a generous way, which is related to the fact that the narratives are based on critical incidents that highlight challenging situations in which their agency also depend on organizational structures. For us, the authors, it is important to show that epistemic injustice arises due to a systemic failure, in which both health care professionals and patients in many situations lack epistemic agency. Even in situations when individual health care professionals are willing to give up their epistemic privilege, this is not sufficient to change the routines and physical environment at care units, especially when it comes to the overall organization of mental health care.

## Data availability statement

The original contributions presented in the study are included in the article/supplementary material, further inquiries can be directed to the corresponding author.

## Ethics statement

Ethical approval was not provided for this study on human participants because this study is an autoethnographic study, which implies that the researchers analyze their own actions. In relation to the subject matter we have followed ethical guidelines and been careful in anonymizing other people and places. Written informed consent for participation was not required for this study in accordance with the national legislation and the institutional requirements.

## Author contributions

LH was responsible for the design of the study and he had the main responsibility for writing the text. LH and MH contributed to data collection. MH collaborated with the LH in the analysis and discussion. All authors contributed to the article and approved the submitted version.

## Funding

Bidragsstiftelsen DHR has funded this publication, that is part of the MOD research programme at Marie Cederschiöld University.

## Conflict of interest

The authors declare that the research was conducted in the absence of any commercial or financial relationships that could be construed as a potential conflict of interest.

## Publisher’s note

All claims expressed in this article are solely those of the authors and do not necessarily represent those of their affiliated organizations, or those of the publisher, the editors and the reviewers. Any product that may be evaluated in this article, or claim that may be made by its manufacturer, is not guaranteed or endorsed by the publisher.
